# Quality Control of Thermally Modified Western Hemlock Wood Using Near-Infrared Spectroscopy and Explainable Machine Learning

**DOI:** 10.3390/polym15204147

**Published:** 2023-10-19

**Authors:** Vahid Nasir, Laurence Schimleck, Farshid Abdoli, Maria Rashidi, Farrokh Sassani, Stavros Avramidis

**Affiliations:** 1 Department of Wood Science Engineering, Oregon State University, Corvallis, OR 97331, USA; laurence.schimleck@oregonstate.edu; 2 Centre for Infrastructure Engineering (CIE), School of Engineering, Design and Built Environment, Western Sydney University, Sydney 2145, Australia; abdolifarshid@gmail.com (F.A.); m.rashidi@westernsydney.edu.au (M.R.); 3 Department of Mechanical Engineering, University of British Columbia, Vancouver, BC V6T 1Z4, Canada; sassani@mech.ubc.ca; 4 Department of Wood Science, University of British Columbia, Vancouver, BC V6T 1Z4, Canada; stavros.avramidis@ubc.ca

**Keywords:** wood modification, thermally treated timber, nondestructive evaluation (NDE), near-infrared (NIR) spectroscopy, feature selection, neural networks, ensemble learning, gradient boosting machine

## Abstract

The quality control of thermally modified wood and identifying heat treatment intensity using nondestructive testing methods are critical tasks. This study used near-infrared (NIR) spectroscopy and machine learning modeling to classify thermally modified wood. NIR spectra were collected from the surfaces of untreated and thermally treated (at 170 °C, 212 °C, and 230 °C) western hemlock samples. An explainable machine learning approach was practiced using a TreeNet gradient boosting machine. No dimensionality reduction was performed to better explain the feature ranking results obtained from the model and provide insight into the critical wavelengths contributing to the performance of classification models. NIR spectra in the ranges of 1100–2500 nm, 1400–2500 nm, and 1700–2500 nm were fed into the TreeNet model, which resulted in classification accuracy values (test data) of 94.35%, 89.29%, and 84.52%, respectively. Feature ranking analysis revealed that when using the range of 1100–2500 nm, the changes in wood color resulted in the highest variation in NIR reflectance amongst treatments. As a result, associated features were given higher importance by TreeNet. Limiting the wavelength range increased the significance of features related to water or wood chemistry; however, these predictive models were not as accurate as the one benefiting from the impact of wood color change on the NIR spectra. The developed framework could be applied to different applications in which NIR spectra are used for wood characterization and quality control to provide improved insights into selected NIR wavelengths when developing a machine learning model.

## 1. Introduction

Thermal modification (TM) is a procedure that involves heating lumber in various atmospheres, such as water, steam, air, vegetable oils, and nitrogen, so that TM timber may be used in outdoor applications. It affects timber’s physical, mechanical, and chemical properties, and changes in these properties are proportional to the intensity of the TM (i.e., temperature and time) [1,2,3,4,5,6]. The primary objective of lumber TM is to improve its dimensional stability against moisture and water. Additionally, it may increase the biological resistance to fungi [7]. However, it may negatively impact the mechanical properties of timber such as the modulus of rupture (MOR), modulus of elasticity (MOE) [8,9,10,11], hardness [12], compressive strength [13], and impact strength [1].

TM degrades hemicelluloses, condenses lignin, and reduces the hydrophilicity of timber [14,15]. Aesthetically speaking, color alterations during modification are significant and are strong functions of temperature and time [11,16,17,18]. Previous research has indicated that the influence of modification temperature on color variations in timber is more significant than that of time. In addition, color change in timber during modification is related to changes in cell wall components (cellulose, hemicellulose, lignin) [19]. Color change in timber is primarily a result of the degradation of hemicelluloses and the oxidation of phenolic compounds [20,21,22] and is dependent on wood pH, moisture content, heating medium, exposure duration, and species. These degradation processes are linked by five fundamental chemistries [23]: hydrolysis, oxidation, dehydration, reduction, and free radical disintegration. 

The aforementioned thermal modifications can result in wood products with different characteristics. To avoid challenges with process control and to ensure that the quality of modified timber is appropriately assessed before placing it on the market under a chain of custody [7], it is crucial to have a thorough understanding of the chemical modifications occurring in the timber during the various thermal modification processes. Nondestructive testing (NDT) is advantageous because it is efficient and economical. It is crucial to investigate the ability of NDT techniques to predict timber’s physical and mechanical properties and classify thermally modified timber (TMT) using NDT techniques [24]. There are several NDT methods for controlling the quality and classification of TMT [7,25,26,27]. Van Blokland et al. [28] used machine learning classification to predict the internal check formation in TMT following natural weathering. It was shown that using the annual ring width in conjunction with the density and initial moisture content or acoustic velocity, the classifier could forecast the check formation with an accuracy of 67%. In addition, [3] reported color-change factors as reliable predictors for MOR, MOE, water absorption, and the swelling of thermally modified timber based on ANN models. 

The wave propagation method was used in a study to identify thermal modification intensity [29]. Accordingly, an acoustic emission sensor and a pair of accelerometers were utilized to classify the TM intensity. The features obtained were subsequently utilized to train neural networks for TMT classification using MLP, GMDH, and linear vector quantization (LVQ). While characteristics obtained from accelerometers, such as stress wave velocity and wood dynamic MOE, demonstrated subpar classification performance, acoustic emission sensory features were influential in classifying TMT. In addition, the performance of the MLP neural network was lower than that of the GMDH and LVQ models. Nasir et al. [30] studied the ThermoWood classification efficacy of artificial neural networks (ANNs), support vector machines (SVMs), and naive Bayes (NB) classifiers. The characteristics of unmodified timber and TMT were determined and evaluated to determine the most suitable set(s) of timber classification characteristics. Consequently, mechanical characteristics, such as the dynamic MOE measured via the stress wave timer test and wood hardness, are the least suitable characteristics, and measuring color offers a precise classification. Both SVMs and the naive Bayes model demonstrated considerably greater accuracy than the ANN model as the latter requires more parameters to be tailored and optimized. According to Nasir et al. [24], the “adaptive neuro-fuzzy inference system” (ANFIS) and the “Group Method of Data Handling” (GMDH) neural network performed better than the multi-layer perceptron (MLP) model for predicting and classifying TMT characteristics. Color measurement was reported to be an important indicator for quality control in TMT [25,31,32]. Schnabel et al. [33] employed color measurement to classify TM hardwood. The challenge of this method may be the non-homogeneous surface color variation [34].

Near-infrared (NIR) spectroscopy has been used in wood characterization and quality control, and visible and NIR spectra (400–2500 nm) were used in a study to classify TMT [24]. The spectra were subjected to dimensionality reduction using linear discriminant analysis (LDA), and the output was used to train an SVM and LVQ neural network. Bachle et al. [35] applied principal component analysis (PCA) and the soft independent modeling of class analogies (SIMCA) for TMT classification using the NIR spectral data. They indicated that the NIR spectrum range impacts the classification accuracy. The knowledge gap in applying NIR spectroscopy for TMT classification stems from the limited research in the literature, the poor interpretability of the machine learning results, and the range of NIR spectra used to develop a classifier. While the use of machine learning applied to NIR spectra for wood characterization and quality control is limited, the developed models could hardly be interpreted. Applying PCA for dimensionality reduction creates a new set of variables lacking clear physical meaning, which makes it challenging to interpret the results [36]. Explainable artificial intelligence (XAI) and using explainable machine learning could address the interpretability of modeling for trustworthiness and confidence [37]. Recently, an explainable machine learning approach was applied to provide insight into the wavelength selection while using NIR spectra for predicting the density and mechanical properties of wood [38]. Yet, there is a gap on using explainable machine learning for better understanding NIR wavelength selection when developing a predictive model using NIR features. Machine learning interpretability methods have been reviewed in the literature [39,40], among which feature importance ranking is a powerful tool for XAI [41].

This study aimed to address the current knowledge gap by providing insight into NIR wavelength selection during the classification of TM wood using an explainable machine learning approach. Accordingly, NIR spectra were used to classify TM Western hemlock (*Tsuga heterophylla*) using a TreeNet gradient boosting machine, a tree-based ensemble learning model. Unlike in the standard practice in the literature, no dimensionality reduction was applied to the spectral data, and the entire NIR dataset was fed into the TreeNet model. The embedded feature selection capability of the model was used for wavelength ranking based on importance level to enhance the interpretability of the results. The top features (wavelengths) were then analyzed using the NIR method to explain the TMT classification. The impact of changing the NIR spectrum range on the classifier’s performance was analyzed, and the results were linked to the wood physics and chemistry based on the band assignment analysis. 

## 2. Materials and Methods

**Figure 1:**
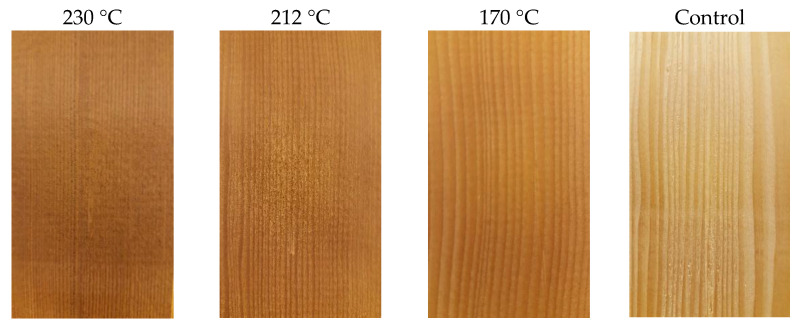
Unmodified and thermally modified western hemlock at 170 °C, 212 °C, and 230 °C, showing the color change in wood by heat treatment intensity.

The experimentation for classifying TM wood using the NIR spectra was according to Nasir et al. [24]. Accordingly, hemlock boards were treated at 170 °C, 212 °C, and 230 °C (Figure 1) using the ThermoWood process with a 2 h holding time at the maximum temperatures. The number of wood samples per treatment was equal to eighty-four, comprising an equal proportion of flat-sawn and quarter-sawn specimens. Eighty-four specimens of untreated wood were also prepared as the control group, resulting in a total sample size equal to 336. Specimens were then conditioned at 20 ± 3 °C and 65 ± 7% relative humidity to reach their equilibrium moisture content (*M_e_*). Spectra were collected using a Quality Spec^®^ Pro Analytical spectrometer (Analytical Spectral Devices Inc., Boulder, CO, USA). Spectrum collection was performed using a fiber-optic probe with an 8 mm diameter perpendicular to the wood surface. Individual spectra comprised 20 independent scans. Also, the spectrum for a sample was the average of six spectra measured from the two transverse surfaces of each sample. Spectra in the range of 1100–2500 nm (1 nm intervals) in reflectance mode were used for machine learning modeling.

Color measurement was also performed on surfaces of wood samples using the CIE L^∗^a^∗^b^∗^ color measuring system, in which the color changes following thermal modification are correlated with the L^∗^a^∗^b^∗^ coordinates. The measurements were performed before and after thermal modification on three spots in the middle and 25 mm from both ends on the surfaces of wood samples (33 × 48 × 214 mm^3^ samples) according to ASTM D2244-16 [42] standard using a Minolta spectrophotometer (model #CM-2600d).

The spectra were classified using a TreeNet gradient boosting machine, a tree-based ensemble learning model. The model could successfully handle NIR spectra without the need for prior dimensionality reduction and was shown to outperform the ANN and convolutional neural network (CNN) for fiber quality prediction using NIR data [43]. Gradient boosting machines have been used in the wood science and technology literature for wood species identification [44] and predicting the properties of wood composites [45]. Decision trees can benefit from ensemble learning algorithms (e.g., bagging, boosting methods). In the boosting method, training of individual decision trees is sequential, wherein the subsequent model improves the performance of the prior one [32]. The advantage of decision tree is its ability to perform despite the presence of noise, missing data, and redundant features [46]. Also, unlike the neural network, gradient boosting machines do not require feature scaling [47]. 

A random portion (defined during the model development) of the training data is used to calibrate a Classification and Regression Tree algorithm (CART) model in TreeNet. Details of CART have been discussed by Steinberg [48], and it is used for predicting the mechanical properties of weathered wood [49]. The maximum number of terminal nodes in the CART should be defined. CART model will be updated based on the loss function, and the update shrinks at a learning rate (defined during the model development). Additional CARTs will be added to improve the error until reaching the maximum number of trees defined in the TreeNet model.

The maximum number of trees in the model was set to 300. Also, the maximum number of terminal nodes was set to 12. Also, the root means square error (RMSE) of the total number of predictors was chosen as the number of predictors for node splitting [50]. Since the model was developed with a small dataset, model validation was performed using a three-fold cross-validation method. Initially, a binary classification was conducted to better understand the important wavelengths used for identifying the thermal treatment intensity. This resulted in the development of six TreeNet models (control vs. 170 °C, control vs. 212 °C, control vs. 230 °C, 170 °C vs. 212 °C, 170 °C vs. 230 °C, 230 °C vs. 212 °C). The learning rate and the subsample fraction were set to 0.01 and 0.3. The predictor elimination technique was used to discover the critical wavelengths (features) with TreeNet multiclass classification. Accordingly, the least essential features were removed to assess their impacts on the model, and the misclassification rate for the test data was computed. The number of trees resulting in the optimal performance of the model was selected. The performance of the selected model was further enhanced using hyperparameter tuning. Accordingly, the model was tested with different values of the learning rate (0.001, 0.010, 0.05, 0.1) and the subsample fraction (0.2, 0.3, 0.4, 0.5) to find the optimal TreeNet model. 

## 3. Results and Discussion

### 3.1. NIR Spectra and Color Features

**Figure 2:**
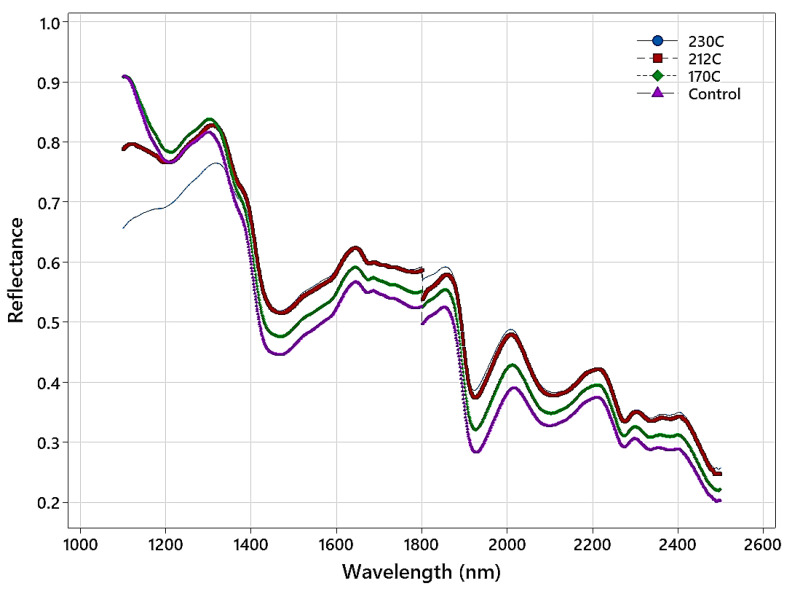
Average NIR spectra from the control and thermally modified samples.

Figure 2 shows the average NIR spectra in the 1100–2500 nm range for the untreated and thermally modified samples. Differences in the NIR spectra of the samples at different temperatures are apparent and arise from color changes in the samples (higher temperature results in darker wood), differences in moisture content amongst the treated samples, and changes in chemistry that become more pronounced at higher temperatures. The NIR spectra of the control samples and those treated at 170 °C are quite similar. Also, they are different from the spectra of samples treated at 212 and 230 °C, with a pronounced downward shift in reflectance as the treatment temperature increased. The largest differences amongst the NIR spectra of the temperature-modified samples are observed in the 1100–1200 nm region, with the spectra of the control and those treated at 170 °C having the highest reflectance, and then in the two higher temperature treatments, whose spectra appear quite different. The difference within the NIR spectrum of the sample treated at 230 °C extends into the 1200–1300 nm region, whereas in this region, the spectra of the other sample groups all appear quite similar.

**Figure 3:**
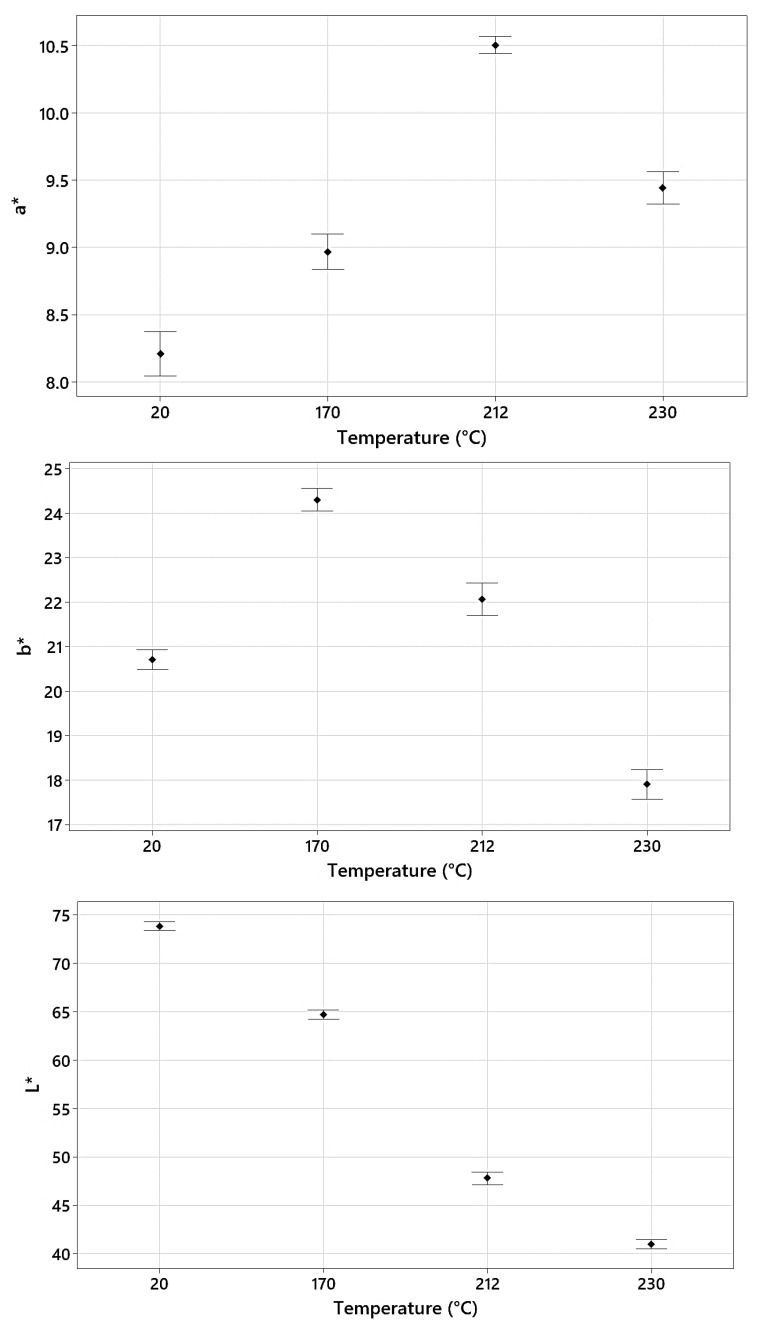
Variation in L*, a*, and b* for the control and thermally modified samples.

Color analysis (L*, a*, and b*) of the control and heat-treated samples (Figure 3) showed that samples progressively became darker (L*) with rises in the modification temperature. However, the values for a* and b* did not change consistently with heat treatment, i.e., they remained the same with the increasing temperature. Instead, a* peaked at 212 °C and b* at 170 °C before decreasing. The largest difference in L* values was observed between 170 °C and 212 °C, with the L* for the two highest temperatures being most similar (likely explained by the relatively small difference (18 °C) between the two treatment temperatures). 

Color change was shown to be correlated with the intensity of thermal modification [11,32,51]. Nourian [52] explained that wood color change following thermal treatment could be explained by colored degradation products from hemicellulose, especially pentosans [53,54], oxidation products [14,55], and extractives [56].

### 3.2. Binary Classification

**Figure 4:**
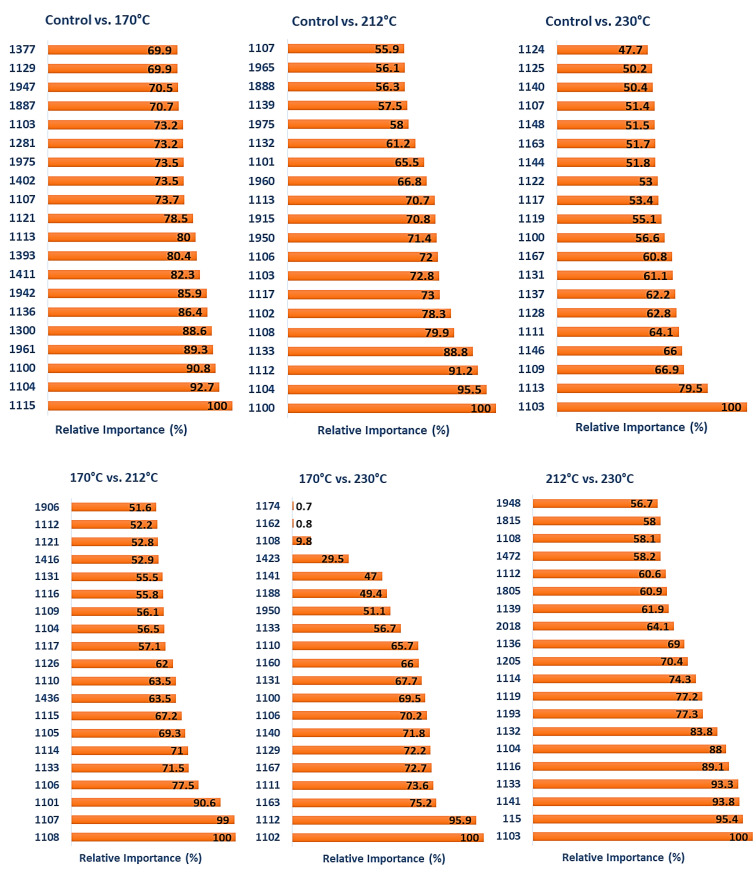
The top 20 wavelengths with the highest relative levels of importance obtained from TreeNet model for binary classification of different timber groups.

The binary classification of the thermally modified samples was completed using TreeNet and NIR spectral data obtained from the surfaces of untreated samples and samples treated at the various modification temperatures. Figure 4 shows the top 20 wavelengths with the highest relative levels of importance obtained from the TreeNet model for the binary classification of different timber groups. The binary classification results for different wood classes are shown in Table 1. 

Analyzing the top 20 crucial features (Figure 4) revealed that the classifications of the untreated (control) samples and those treated at temperatures of 170 °C and 212 °C were very similar. Amongst the features utilized in classification, those in the range of 1100–1140 nm were frequently employed (nine and fourteen of the top twenty for control vs. 170 °C and control vs. 212 °C, respectively), and the appearance of features from this region of the NIR spectrum was related to the significant spectral differences observed in Figure 2 and the increasing darkness of the samples (Figure 3). The greater reliance of the untreated versus 212 °C classification on this region can be related to the greater darkness of these samples compared to those treated at 170 °C. Thermal modification decreases the equilibrium moisture content in treated samples [1], and for both classifications, water-related features were also important. These included 1942, 1947, 1961, and 1975 nm for the binary classification of control vs. 170 °C and 1915, 1959, 1960, 1965, and 1975 nm for the binary classification of control vs. 212 °C (1916–1942 nm is assigned to O-H asymmetric stretch + O-H deformation and 1980 nm = O-H stretch + O-H deformation in water). A nearby feature at 1887/1888 nm was also utilized in the classification but had no band assignment based on the summary, presented by Schwanninger et al. [57], of the NIR features of wood and its components. The remaining important wavelengths for the control vs. 170 °C included 1377 nm and 1393 nm (bands at 1370 nm and 1386 nm arise from the 1st overtone (OT) C-H stretch and C-H deformation vibrations in hemicellulose and other wood constituents) and there were features at 1402 and 1411 nm; nearby bands in this region had arisen from the 1st OT O-H stretch vibrations in lignin/extractives (1410 nm) and water (1414 nm). Two features (1281 and 1300 nm) utilized in the control vs. 170 °C were unassigned.

For the control vs. 230 °C, the classification relied only on features in the 1100–1167 nm range. The utilization of wavelengths in this region can be directly related to the large spectral and darkness (L*) differences between the control samples and those modified at 230 °C (Figure 1 and Figure 3). In terms of treatment temperature, the difference between 212 °C and 230 °C is relatively small, but the impact on NIR reflectance is quite significant and is sufficient to wholly overshadow any differences owing to wood chemistry and water (moisture) content elsewhere in the NIR spectral range, differences that were important for the control vs. 170 °C and 212 °C classifications.

The binary classification of samples modified at 170 °C vs. 212 °C relied heavily on the range of 1100–1133 nm, with seventeen features from this region being utilized. A significant shift in NIR reflectance in this region was observed (Figure 2) between the two modification temperatures, explaining the importance of features in this region. The further darkening of the samples at 212 °C was pronounced compared to those at 170 °C, and the largest difference in L* values was observed between these two temperatures (Figure 3). The remaining features were again related to bands associated with wood chemistry and water. These included 1416 nm (1414 nm = water 1st OT O-H stretch, 1417 nm = lignin 1st OT C-H stretch and C-H bend), 1436 nm (1435–1438 nm = water 1st OT O-H stretch, 1440 nm = lignin 1st OT C-H stretch and C-H deformation) and 1906 nm (1907 and 1910 nm = hemicellulose 2nd OT C=O stretch). 

The classification of samples treated at 170 °C vs. 230 °C was very similar to that treated at 170 °C versus 212 °C in terms of the features utilized, with eighteen features in the range of 1100–1174 nm selected. The remaining features utilized included 1423 nm (1428 nm = cellulose and water 1st OT O-H stretch) and 1950 nm (1916–1942 nm = water O-H asymmetric stretch + O-H deformation). While both classifications rely heavily on spectral shifts in the region of 1100–1200 nm, the utilization of water and chemistry-related features suggest important wood chemistry changes at higher modification temperatures and a change (decrease) in the equilibrium moisture content of samples treated at 212 °C and 230 °C compared to those treated at 170 °C.

The final classification compared samples modified at 212 °C and 230 °C. Wavelengths close to 1100 nm again dominated the classification, with 14 features in the range of 1103–1193 nm selected. The lower number of features from this region, compared to other modification temperature classifications, may be attributed to similar L* values for samples treated at 212 °C and 230 °C. The slight change in emphasis to other regions of the NIR spectrum resulted in identifying features mainly related to cellulose, lignin, and hemicellulose. These included 1205 nm, which occurs close to two identified bond vibrations, namely 1188–1195 nm (lignin and cellulose 2nd OT C-H stretch) and 1212–1225 nm (cellulose 2nd OT C-H stretch); 1472 nm (1471 nm = hemicellulose 1st OT O-H stretch), 1805 and 1815 nm (1811 nm, unknown bond vibration but assigned to lignin, 1820 nm = cellulose O-H stretch + 2nd OT C-O stretch); and 2456 nm (2461 nm = carbohydrate C-H stretch + C-C stretch). The remaining features included those at 1948 nm, which, as already noted, occurs very close to a region assigned to the O-H stretch and deformation vibrations in water, and 2018 nm, which is unassigned. Samples treated at 212 °C and 230 °C likely have similar equilibrium moisture contents, which would explain the low importance of water-related features in the classification.

### 3.3. Multiclass Classification (1100–2500 nm)

**Figure 5:**
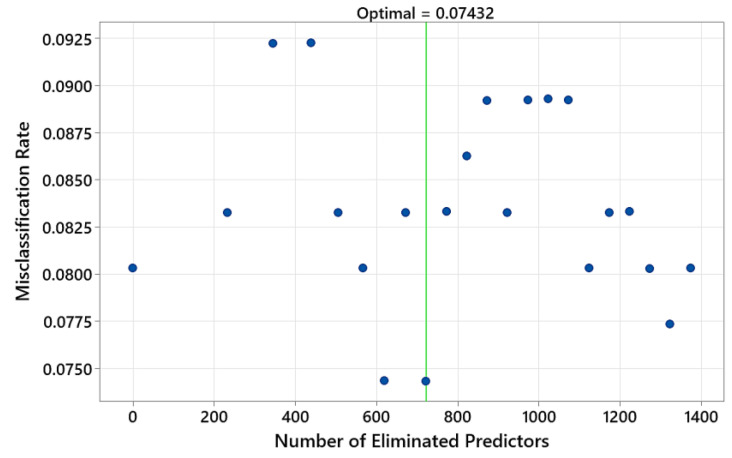
Predictor elimination analysis for finding the number of predictors in the model yielding the minimum misclassification rate on test data (input NIR range: 1100–2500 nm).

Following the binary classification and understanding of the mechanism of the wavelength selection, the spectra in the 1100–2500 nm range were fed into the TreeNet model for multiclass classification. Predictor elimination analysis was performed to find the number of predictors in the model yielding the minimum misclassification rate on the test data. The analysis found 722 wavelengths (predictors) to be eliminated from the model. The remaining 679 wavelengths resulted in a model with a minimum misclassification rate of 0.074 on test data (Figure 5). 

Hyperparameter tuning was performed on this model to find the optimal combination of learning rate and subsample fraction. Table 2 shows that for a learning rate of 0.1 and subsample fraction of 0.4 (and a fixed maximum terminal node equal to 12), the minimum misclassification rate will drop, reaching a minimum value of 0.056. 

**Figure 6:**
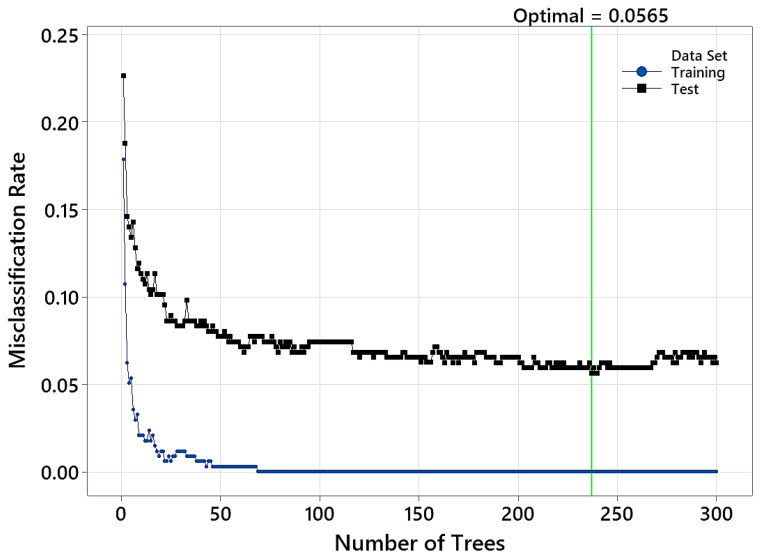
Variation in the misclassification rate versus the number of trees in the TreeNet model (input NIR range: 1100–2500 nm).

The variation in the misclassification rate versus the number of trees in the TreeNet model is shown in Figure 6. Optimal performance is achieved when having a model with 237 trees. Table 3 shows the confusion matrix and model accuracy. The overall classification accuracy on the test data is 94.35%. Also, it is seen that the highest confusion between the classes belonged to the control group, in which ten samples were misclassified as having been treated at 170 °C. Misclassification may have occurred because these samples were slightly darker than other untreated samples and were, therefore, more similar to samples treated at 170 °C.

**Figure 7:**
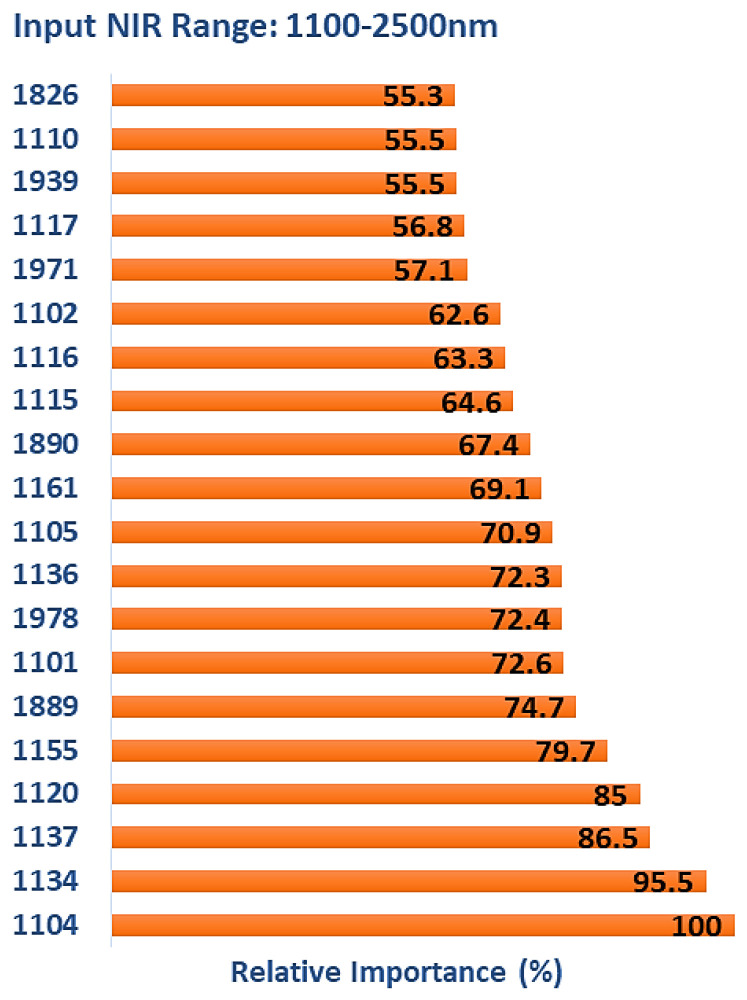
The top 20 wavelengths with the highest relative levels of importance obtained from TreeNet model after multiclass classification (input NIR range: 1100–2500 nm).

Out of the 679 input wavelengths, only one was unimportant, and the rest contributed to model performance. Figure 7 lists the top 20 wavelengths with the highest relative levels of importance obtained from the TreeNet model after multiclass classification. The majority of features selected were consistent with the binary classifications, with 14 in the range of 1101–1161 nm, the region where a significant shift in NIR reflectance values occurs as treatment temperature increases (Figure 2). Four of the remaining features occurred in regions also observed for the binary classifications and included 1939, 1971, and 1978 nm (1916–1942, and 1980 nm = water-associated vibrations) and 1826 nm (1820 and 1830 nm = cellulose). The features at 1889 and 1890 nm are unassigned, with the closest assigned wavelengths being 1907 and 1910 nm (hemicellulose 2nd OT C=O stretch).

### 3.4. Multiclass Classification (1400–2500 nm)

**Figure 8:**
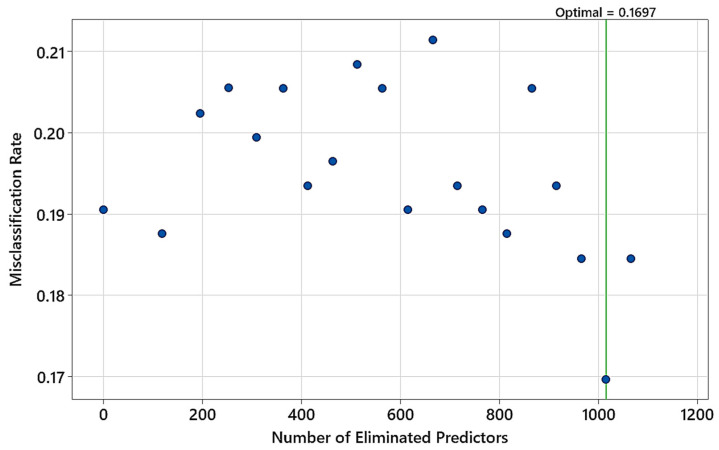
Predictor elimination analysis for finding the number of predictors in the model yielding the minimum misclassification rate on test data (input NIR range: 1400–2500 nm).

To study the impact of the NIR spectrum range on the performance of the TreeNet model, and how it may impact wavelength selection by the machine learning model, the spectral range was limited to 1400–2500 nm for multiclass classification. Predictor elimination resulted in removing more than 1000 wavelengths and leaving only 86 features as predictors (Figure 8). The minimum misclassification rate of the model was 0.1697 on the test data. 

Similar to what was described in the previous section, hyperparameter tuning was performed. According to Table 4, the model with the lowest misclassification rate on the test data had a learning rate of 0.1 and a subsample fraction of 0.3. This resulted in the initial misclassification rate (0.1697) dropping to 0.1071. 

**Figure 9:**
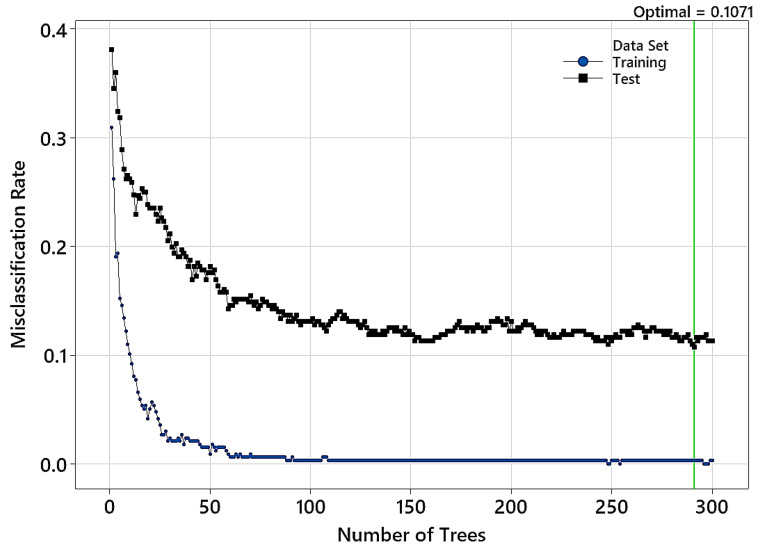
Variation in the misclassification rate versus the number of trees in the TreeNet model. (Input NIR range: 1400–2500 nm).

Figure 9 shows how the misclassification rate changes with the number of trees in the TreeNet model. The misclassification rate almost stays at its optimal value after adding 88 trees. However, this rate is minimal for the test data containing 291 trees in TreeNet. The confusion matrix and model accuracy are listed in Table 5. The overall classification accuracy values on the train and test data are 99.7% and 94.35%, respectively. Similar to what was noted in the previous modeling (NIR range = 1100–2500 nm), the highest confusion was again between the control group and samples treated at 170 °C.

**Figure 10:**
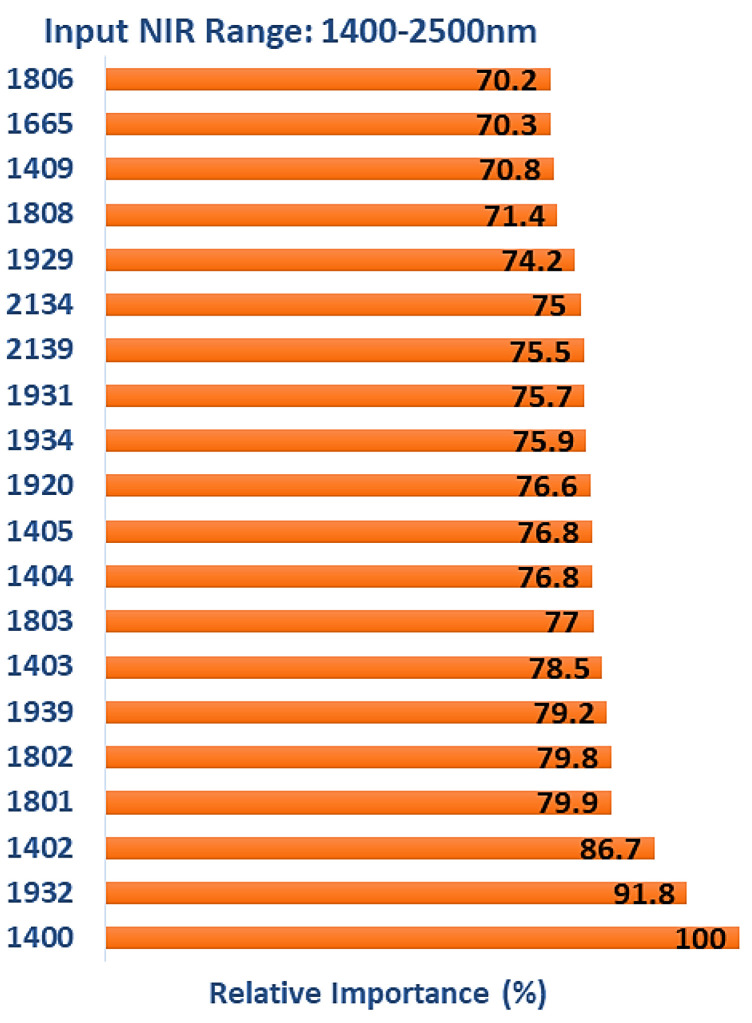
The top 20 wavelengths with the highest relative levels of importance obtained from TreeNet model after multiclass classification (input NIR range: 1400–2500 nm).

It can be seen that the overall classification accuracy of the model (test data) trained with spectra in the range of 1400–2500 nm (89.29%) is lower than when trained with NIR spectra in the range of 1100–2500 nm (94.35%), indicating the importance of features (reflectance shifts related to color changes) in the range of 1100–1200 nm. The important wavelengths contributing to the model performance could explain the weaker classification. Analyzing the critical features indicates that all of the 86 input wavelengths were considered essential and contributed positively to the model’s performance. Figure 10 lists the top 20 wavelengths with the highest relative levels of importance obtained from TreeNet after multiclass classification. Limiting the wavelength range saw features that were associated with either water or wood chemistry being utilized. Six of the water-related features occurred in the region of 1920–1934 nm, and six in the region of 1400–1409 nm (1414 nm = water). The region of 1400–1409 nm is also related to wood chemistry (1410 nm = lignin/extractives). Other chemistry-related features included 1665 nm (hemicellulose 1st OT C-H stretch (1666 nm) or extractives 1st OT C_ar_-H stretch (1668 nm)), five in the range of 1801–1806 nm (1793 nm = cellulose 1st OT O-H stretch, 1811 nm = lignin) and 2134/2139 nm (2134 nm = hemicellulose tentatively assigned to C-H stretch + C=O stretch and lignin/extractives C_ar_-H stretch + C=O stretch).

### 3.5. Multiclass Classification (1700–2500 nm)

**Figure 11:**
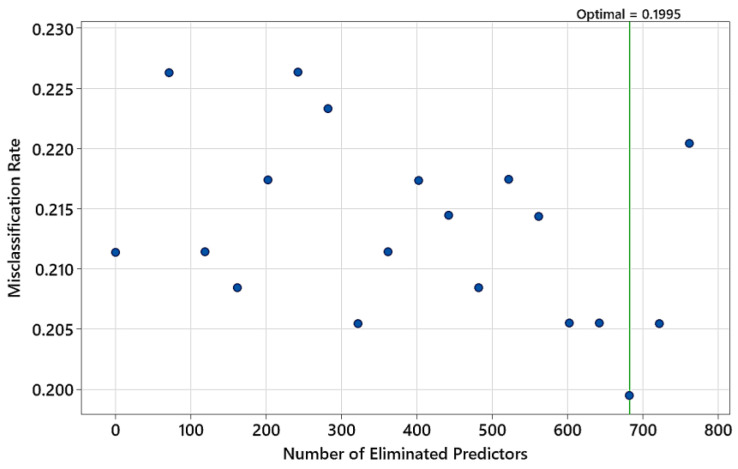
Predictor elimination analysis for finding the number of predictors in the model yielding the minimum misclassification rate on test data (input NIR range: 1700–2500 nm).

Finally, the TreeNet model was trained with the NIR spectra in the 1700–2500 nm range. Predictor elimination resulted in the model having 119 wavelengths (features) as predictors (Figure 11). The model minimum misclassification rate on the test data was 0.1995. 

**Figure 12:**
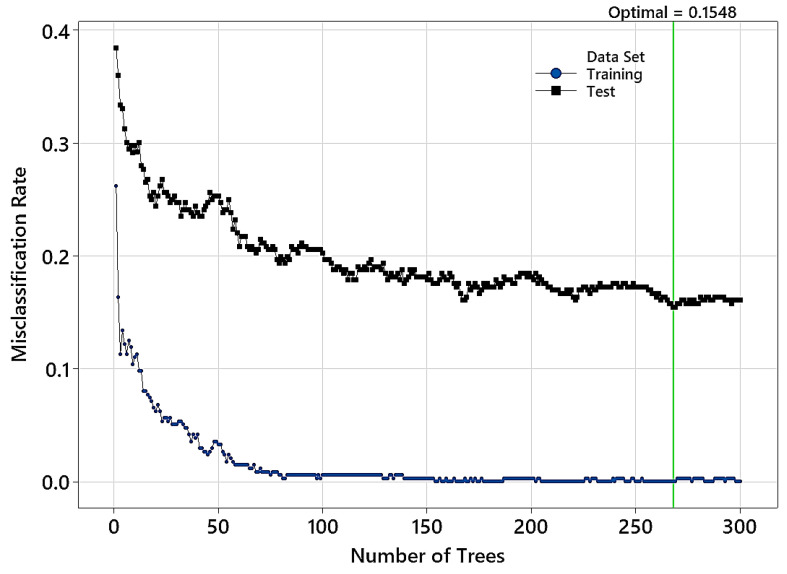
Variation in the misclassification rate versus the number of trees in the TreeNet model (input NIR range: 1700–2500 nm).

Hyperparameter tuning resulted in an optimal model with a misclassification rate of 0.1548 on the test data. The chosen model’s learning rate and subsample fraction were 0.05 and 0.4, respectively (Table 6). Figure 12 shows that the optimal model had 268 trees in its structure. Analyzing the confusion matrix (Table 7) indicates a reduction in the classification accuracy for all groups of wood samples. The highest misclassification rate belonged to wood treated at 170 °C (accuracy of 79.76%), followed by the control group (accuracy of 85.71%). The highest accuracy was for wood treated at 230 °C (89.29%).

**Figure 13:**
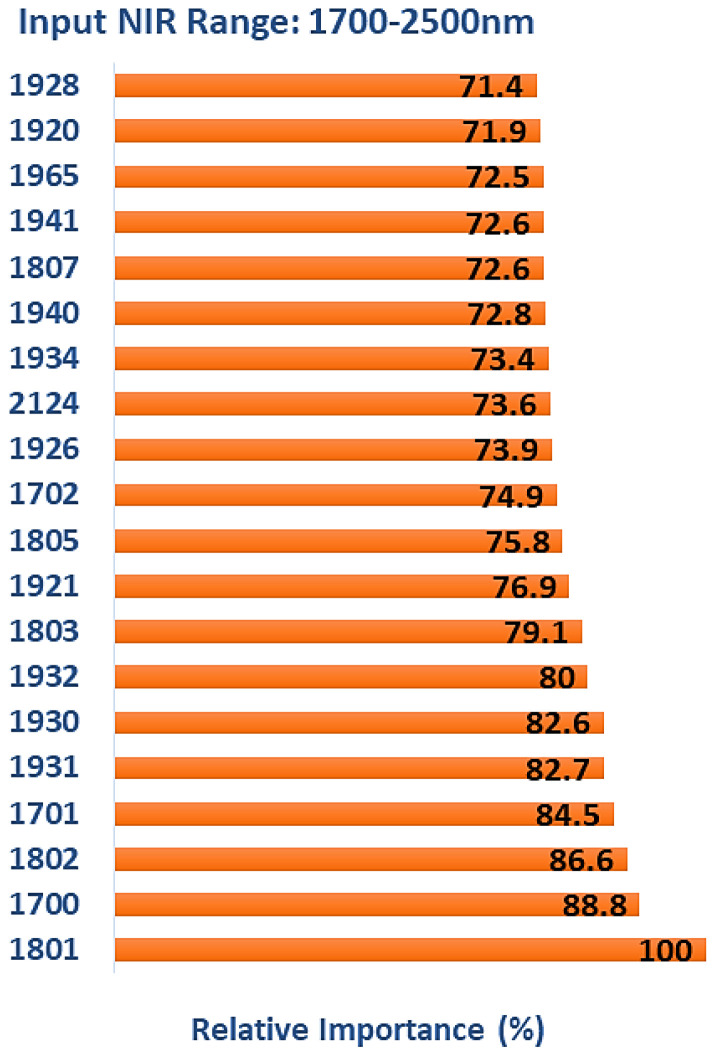
The top 20 wavelengths with the highest relative levels of importance obtained from TreeNet model after multiclass classification (input NIR range: 1700–2500 nm).

The model’s overall accuracy was 84.52% on the test data, which was lower than the accuracy values obtained when TreeNet was trained with NIR spectra in the range of 1100–2500 nm and 1400–2500 nm. To explain this reduction in the model’s performance, the results of wavelength selection and feature ranking were used for further analysis. Figure 13 lists the top 20 features with the highest relative levels of importance obtained from the TreeNet model after multiclass classification. The further restriction of the wavelength range used for classification resulted in a greater emphasis on water-related features in the 1916–1942 nm range (ten in total plus 1965 nm). The region of 1801–1807 nm (cellulose/lignin) was again important, with five features, as was 2134 nm (hemicellulose and lignin/extractives). The remaining features (three) were in the region of 1700–1702 nm, which is associated with the 1st OT O-H stretch vibrations in lignin (1698 nm), cellulose (1703 nm), and hemicellulose (1705 nm).

This study was performed on a small dataset, which could be expanded for future studies. TreeNet does not require prior feature scaling and selection or dimensionality reduction [50]. Also, the ability to handle missing data and resistance to outliers in predictors or the target parameter [50] makes it an effective method for analyzing NIR spectral data. Grinsztajn et al. [58] showed the superior performance of gradient boosting machines over neural networks on medium-sized data (~10,000 samples). Future studies could focus on benchmarking the thermally modified wood classification using different machine learning or deep learning models. Finally, explainable machine learning approaches, such as employing feature importance ranking when developing a machine learning model, could be used to analyze NIR spectra for predicting different physical and mechanical properties of thermally modified wood. 

## 4. Conclusions

This study’s explainable machine learning approach provided insight into the important wavelengths contributing to the classification of thermally modified timber. Gradient boosting machines could successfully identify the intensity of thermal modification without the need to perform dimensionality reduction on the NIR spectra. The best classification accuracy (94.35% on the test data) was obtained when spectra in the 1100–2500 nm range were used to train the model. The restriction of the wavelength range lowered the accuracy of the classifiers. Feature ranking was performed, and the important wavelengths were analyzed. For the spectra in the 1100–2500 nm range, the majority of selected features were consistent with the binary classifications in the 1101–1161 nm range, where a significant shift in NIR reflectance values occurred as the treatment temperature increased, resulting in increased darkness. In other words, changes in wood color cause the greatest variation in NIR reflectance amongst treatments, and as a result, features associated with that are chosen by the expert model for classification. Limiting the wavelength range to 1400–2500 nm increased the importance of features that were either associated with water or wood chemistry, and the further restriction of the wavelength range to 1700–2500 nm resulted in a greater emphasis on water-related features. The technique could be applied to other applications in which NIR spectroscopy is used for wood characterization and quality control and provides improved insight into how the expert model utilizes NIR wavelengths.

## Figures and Tables

**Table 1:** The results of binary classification for different timber classes.

Statistics	Control vs. 170 °C		Control vs. 212 °C		Control vs. 230 °C		170 °C vs. 212 °C		170 °C vs. 230 °C		212 °C vs. 230 °C	
	Training	Test	Training	Test	Training	Test	Training	Test	Training	Test	Training	Test
True positive rate (Sensitivity) %	100.00	90.48	100.00	98.81	100.00	100.00	100.00	100.00	100.00	100.00	97.62	96.43
False positive rate (type I error) %	3.57	8.33	1.19	0.00	0.00	0.00	0.00	1.19	1.19	0.00	2.38	2.38
False negative rate (type II error) %	0.00	9.52	0.00	1.19	0.00	0.00	0.00	0.00	0.00	0.00	2.38	3.57
True negative rate (specificity) %	96.43	91.67	98.81	100.00	100.00	100.00	100.00	98.81	98.81	100.00	97.62	97.62

**Table 2:** Finding the optimal model (bolded) with minimum misclassification for multiclass classification based on features in the range of 1100–2500 nm.

Model	Optimal Number of Trees	Misclassification Rate	Learning Rate	Subsample Fraction	Maximum Terminal Nodes
1	173	0.175	0.001	0.2	12
2	283	0.101	0.010	0.2	12
3	115	0.080	0.050	0.2	12
4	140	0.071	0.100	0.2	12
5	126	0.136	0.001	0.3	12
6	299	0.068	0.010	0.3	12
7	239	0.059	0.050	0.3	12
8	272	0.065	0.100	0.3	12
9	166	0.113	0.001	0.4	12
10	253	0.080	0.010	0.4	12
11	245	0.062	0.050	0.4	12
**12**	**237**	**0.056**	**0.100**	**0.4**	**12**
13	96	0.113	0.001	0.5	12
14	287	0.080	0.010	0.5	12
15	246	0.065	0.050	0.5	12
16	208	0.068	0.100	0.5	12

**Table 3:** Confusion matrix and model accuracy for multiclass classification based on features in the range of 1100–2500 nm.

Wood Class	Count	Predicted Class (Training)					Predicted Class (Test)				
		230°C	212 °C	170 °C	Control	Correct %	230 °C	212 °C	170 ° C	Control	Correct %
230 °C	84	84	0	0	0	100.00	81	3	0	0	96.43
212 °C	84	0	84	0	0	100.00	3	81	0	0	96.43
170 °C	84	0	0	84	0	100.00	0	0	81	3	96.43
Control	84	0	0	0	84	100.00	0	0	10	74	88.10
All	336	84	84	84	84	100.00	84	84	91	77	94.35

**Table 4:** Finding the optimal model (bolded) with minimum misclassification for multiclass classification based on features in the range of 1400–2500 nm.

Model	Optimal Number of Trees	Misclassification Rate	Learning Rate	Subsample Fraction	Maximum Terminal Nodes
1	113	0.330	0.001	0.2	12
2	254	0.226	0.010	0.2	12
3	254	0.142	0.050	0.2	12
4	295	0.128	0.100	0.2	12
5	293	0.270	0.001	0.3	12
6	270	0.169	0.010	0.3	12
7	287	0.130	0.050	0.3	12
**8**	**291**	**0.107**	**0.100**	**0.3**	**12**
9	66	0.223	0.001	0.4	12
10	254	0.160	0.010	0.4	12
11	288	0.125	0.050	0.4	12
12	198	0.121	0.100	0.4	12
13	297	0.214	0.001	0.5	12
14	298	0.148	0.010	0.5	12
15	219	0.124	0.050	0.5	12
16	193	0.116	0.100	0.5	12

**Table 5:** Confusion matrix and model’s accuracy for multiclass classification based on features in the range of 1400–2500 nm.

Wood Class	Count	Predicted Class (Training)					Predicted Class (Test)				
		230 °C	212 °C	170 °C	Control	Correct %	230 °C	212 °C	170 °C	Control	Correct %
230 °C	84	84	0	0	0	100.00	77	4	3	0	91.67
212 °C	84	1	83	0	0	98.81	6	76	2	0	90.48
170 °C	84	0	0	84	0	100.00	0	5	73	6	86.90
Control	84	0	0	0	84	100.00	0	0	10	74	88.10
All	336	85	83	84	84	99.70	83	85	88	80	89.29

**Table 6:** Finding the optimal model (bolded) with minimum misclassification for multiclass classification based on features in the range of 1700–2500 nm.

Model	Optimal Number of Trees	Misclassification Rate	Learning Rate	Subsample Fraction	Maximum Terminal Nodes
1	165	0.375	0.001	0.2	12
2	295	0.267	0.010	0.2	12
3	282	0.193	0.050	0.2	12
4	265	0.163	0.100	0.2	12
5	290	0.318	0.001	0.3	12
6	296	0.199	0.010	0.3	12
7	294	0.157	0.050	0.3	12
8	152	0.157	0.100	0.3	12
9	284	0.256	0.001	0.4	12
10	300	0.190	0.010	0.4	12
**11**	**268**	**0.154**	**0.050**	**0.4**	**12**
12	293	0.160	0.100	0.4	12
13	240	0.250	0.001	0.5	12
14	292	0.205	0.010	0.5	12
15	155	0.175	0.050	0.5	12
16	222	0.163	0.100	0.5	12

**Table 7:** Confusion matrix and model accuracy for multiclass classification based on features in the range of 1700–2500 nm.

Actual Class	Count	Predicted Class (Training)					Predicted Class (Test)				
		230 °C	212 °C	170 °C	Control	Correct %	230 °C	212 °C	170 °C	Control	Correct %
230 °C	84	84	0	0	0	100.00	75	7	2	0	89.29
212 °C	84	0	84	0	0	100.00	9	70	5	0	83.33
170 °C	84	0	0	84	0	100.00	2	9	67	6	79.76
Control	84	0	0	0	84	100.00	0	1	11	72	85.71
All	336	84	84	84	84	100.00	86	87	85	78	84.52

## Data Availability

Not applicable.
